# A food poisoning caused by ST7 *Staphylococcal aureus* harboring *sea* gene in Hainan province, China

**DOI:** 10.3389/fmicb.2023.1110720

**Published:** 2023-03-16

**Authors:** Yahui Guo, Xiaojie Yu, Jixiao Wang, De Hua, Yuanhai You, Qingbo Wu, Qinglong Ji, Jianzhong Zhang, Liefei Li, Yuan Hu, Zhonghui Wu, Xiaoyue Wei, Lianqun Jin, Fanliang Meng, Yuhua Yang, Xiaofeng Hu, Lijin Long, Songnian Hu, Heyuan Qi, Juncai Ma, Wenwen Bei, Xiaomei Yan, Haibin Wang, Zilong He

**Affiliations:** ^1^Baotou Medical College, Inner Mongolia University of Science and Technology, Baotou, China; ^2^Chinese Center for Disease Control and Prevention, National Institute for Communicable Disease Control and Prevention, Beijing, China; ^3^Beijing Chaoyang District Center for Disease Control and Prevention, Beijing, China; ^4^Hainan Provincial Center for Disease Control and Prevention, Haikou, China; ^5^Chinese Academy of Inspection and Quarantine, Beijing, China; ^6^Chinese PLA Center for Disease Control and Prevention, Beijing, China; ^7^State Key Laboratory of Microbial Resources, Institute of Microbiology, Chinese Academy of Sciences, Beijing, China; ^8^Microbial Resource and Big Data Center, Institute of Microbiology, Chinese Academy of Sciences, Beijing, China; ^9^Beijing Advanced Innovation Center for Big Data-Based Precision Medicine, Interdisciplinary Innovation Institute of Medicine and Engineering, School of Engineering Medicine, Beihang University, Beijing, China

**Keywords:** staphylococcus food poisoning, sequence type 7, whole-genome sequencing, enterotoxin A, prophage, plasmids

## Abstract

ST7 *Staphylococcus aureus* is highly prevalent in humans, pigs, as well as food in China; however, staphylococcal food poisoning (SFP) caused by this ST type has rarely been reported. On May 13, 2017, an SFP outbreak caused by ST7 *S. aureus* strains occurred in two campuses of a kindergarten in Hainan Province, China. We investigated the genomic characteristics and phylogenetic analysis of ST7 SFP strains combined with the 91 ST7 food-borne strains from 12 provinces in China by performing whole-genome sequencing (WGS). There was clear phylogenetic clustering of seven SFP isolates. Six antibiotic genes including *blaZ, ANT (4′)-Ib, tetK, lnuA, norA,* and *lmrS* were present in all SFP strains and also showed a higher prevalence rate in 91 food-borne strains. A multiple resistance plasmid pDC53285 was present in SFP strain DC53285. Among 27 enterotoxin genes, only *sea* and *selx* were found in all SFP strains. A ФSa3int prophage containing type A immune evasion cluster (*sea, scn, sak*, and *chp*) was identified in SFP strain. In conclusion, we concluded that this SFP event was caused by the contamination of cakes with ST7 *S. aureus*. This study indicated the potential risk of new emergencing ST7 clone for SFP.

## Introduction

Food-borne disease (FBD) caused by microorganisms is a major concern for food safety and it is a global health problem that is harmful to humans. *Staphylococcus aureus* an ubiquitous Gram-positive bacterium and a common causative pathogen of food poisoning (Hennekinne et al., 2012). A total of 314 outbreaks of staphylococcal food poisoning (SFP) were reported in China between 2011 and 2016, involving 5,196 cases and 1 death (Liu et al., 2018). In 2020, the National Foodborne Disease Outbreak Surveillance System in China reported 4,662 outbreaks with confirmed etiology, of which bacterial pathogens were the most common cause of illnesses (41.7%) and *S. aureus* ranked third among the causative bacteria (75 outbreaks and 954 illnesses) (Li et al., 2021). The incubation period of SFP is 0.5–8 h, and the common symptoms are nausea and vomiting, with or without abdominal pain, dizziness, diarrhea, shivering, general weakness, and moderate fever (Hennekinne et al., 2012).

*Staphylococcus aureus* can produce different Staphylococcal enterotoxins (SEs)and SE-like (SEl) toxins. It was reported less than 1 μg of SE can cause SFP (Pinchuk et al., 2010). The most common SEs are SEA, SEB, SEC, SED and SEE, accounting for approximately 95% of SFP outbreaks (Bastos et al., 2017). Most of the SEs and SEl toxins are located on the mobile genetic elements (MGEs) (such as plasmids, prophages, genomic islands, and pathogenicity islands) or next to the staphylococcal cassette chromosome (Argudin et al., 2010). Among them, SEA and SEP are carried by prophages (Argudin et al., 2010).

ST7 *S. aureus* is an important circulating clone in China. ST7 is one of the most commonly isolated STs from the nasal swabs of healthy individuals and occupational livestock workers in China (Yan et al., 2015; Ye et al., 2015). ST7 is also the dominant type in healthy and diseased pigs, and environment (pigpen gates, soil, and ground), followed by ST398 and ST9 in China (Yan et al., 2014; Zhou et al., 2020). ST7 strains can cause a variety of diseases both in animals and humans. ST7 clones are common cause of bovine mastitis (Li et al., 2017). Among the human-related diseases, ST7 clones commonly cause skin and soft tissue infections, bacteremia, pneumonia, and musculoskeletal infection, especially in children (Gu et al., 2016; Li et al., 2018; Wu et al., 2018). Most importantly, ST7 is the dominant type in a variety of foods, such as ready-to-eat foods and vegetables (Liao et al., 2018; Rong et al., 2018).

At present, ST6, ST943, ST5, ST59, ST81, and ST8 were the predominant types of *S. aureus* that caused food poisoning outbreaks in many countries (Cha et al., 2006; Suzuki et al., 2014; Li et al., 2018; Chen and Xie, 2019; Lv et al., 2021). Untill now, outbreaks of ST7 food poisoning have been only reported in Shijiazhuang and Guangzhou in China (Lv et al., 2021; Zhou et al., 2022). On May 13, 2017, an SFP outbreak caused by ST7 *S. aureus* strains occurred in two campuses of a kindergarten in Hainan Province, China, involving 26 children and one adult. ST7, as a new emerging SFP clone, is not well characterized up to now. In this study, we aimed to investigate the genomic characteristics of these ST7 strains, by using whole-genome sequencing (WGS). In particular, we analyzed the SFP strains for virulence, drug resistance, prophages, plasmid, and defense systems. Moreover, we conducted phylogenetic analysis of these SFP strains along with the 91 ST7 strains isolated from food in 12 provinces of China.

## Materials and methods

### Epidemiological investigation

On May 13, 2017, an outbreak of food poisoning in campuses A and B of a kindergarten in the Hainan Province, China was investigated. The outbreak involved 26 children and one adult. The symptoms had appeared after consumption of breakfast. The most common symptoms were vomiting (27/27, 100%), followed by periumbilical abdominal pain (14/27, 51.85%), and fever (3/27, 11.11%). The incubation period for symptoms was 1–3 h. The cakes purchased from a cake shop were suspected of being related to the SFP. The leftover food and anal swabs of patients were collected for bacterial isolation. All samples were processed based on *S. aureus* isolation methods for foods according to the national standards in China (GB/T 4789.10–2017). Five *S. aureus* strains were isolated from leftover cake and lean porridge in campus A, the cream from cake shop, and leftover cake in campus B, while two strains from patients were isolated from campus A.

### Bacterial collection

Ninety-one food-borne ST7 *S. aureus* strains were collected from 12 provinces of China between 2006 and 2019 (Supplementary Figure S1). All the strains in the study were confirmed by PCR detection for *nuc* and *mecA* (Merlino et al., 2002).

### Antimicrobial susceptibility testing

The minimum inhibitory concentrations (MICs) of cefoxitin, linezolid, clindamycin, erythromycin, doxycycline, gentamicin, penicillin, rifampicin, sulfamethoxazole, tigecycline, ciprofloxacin, tetracycline, vancomycin, fusidic acid, quinupristin/dalfopristin, daptomycin, teicoplanin, florfenicol, tiamulin, and nitrofurantoin for 7 SFP strains were determined by microdilution method. *S. aureus* ATCC29213 was used as the quality control strain. The MICs values were interpreted according to Clinical and Laboratory Standard Institute (CLSI) Performance Standards for Antimicrobial Susceptibility Testing (31^st^ edition).

### DNA extraction, genome sequencing, assembly, and annotation

Genomic DNA was extracted from pure cultures using a commercial kit (QIAGEN, Germany) and quantified by Qubit 4.0 (USA Invitrogen ABI). Genomic DNA was sequenced using Illumina NovaSeq PE150 by the Beijing Novogene Bioinformatics Technology Co. Ltd. All sequences were preprocessed by READFQ V10 (Chen et al., 2018) to delete data with mass value ≤20. Clean data were assembled using SPAdes V3.13 (Bankevich et al., 2012), from which only contigs greater than 500 bp in length were selected for further analysis. The Nanopore Oxford MinION platform was used for three strains, i.e., DC53285, DC53206, and DC52998 (You et al., 2018). Unicycler v0.5.0 (Wick et al., 2017) was used for genome assembly based on ONT long reads and Illumina short reads. All genomes were annotated by Prokka V1.14.5 (Seemann, 2014). The closed genome was confirmed by PCR (Supplementary Table S4).

### Phylogenetic analysis

The genomes of seven SFP strains, 91 food-borne strains, and seven ST7 published sequences from NCBI (Supplementary Table S1) were included in the phylogenetic analysis. DC51277 (CC7-ST943) was used as an outgroup. The core genome was extracted by Roary 3.13.0 (Page et al., 2015). Maximum-likelihood phylogenetic trees were constructed using RAxML V8.2.12 (Stamatakis, 2014) based on 2,387 core genes (shared by >99% of *S. aureus* isolates) and the evolutionary tree was visualized by ITOL^1^ (Letunic and Bork, 2019).

### Multilocus sequencing typing (MLST), detection of virulence, resistance, restriction-modification genes (R-M), prophage, and plasmid

MLST was performed by PubMLST^2^ website. VFDB and CARD libraries were downloaded to construct the local database, and the search within blastp was conducted with parameters of >80% identity and >80% coverage. According to the copy number of virulence and resistance genes, heatmap was drawn using the heatmap package of R V4.1.2. (Chan, 2018). To better assess the carriage rate of virulence genes, our data were compared with the data of a previously published manuscript (Slingerland et al., 2020), in which genome of 10,288 *S. aureus* strains were included. Statistical analysis was performed by Chi-squared test for independence using SPSS Statistics 25.0 (IBM). *p* values <0.05 were considered indicative of statistical significance.

R-M genes were searched on Restriction-Modification Finder 1.1.^3^ *hsdM* and *hsdS* were verified by PCR (Supplementary Table S4). CRISPR-cas genes were searched on CRISPRCas Finder (Couvin et al., 2018).

The prophage structure of four strains was compared, including one SFP strain (DC53285) and three non-SFP strains which carried *sea* (DC53206, DC52998, and DC52929). Prophages and plasmids were identified by PHASTER^4^ and PlasmidFinder.^5^ The prophage and plasmid nucleotide sequences were compared with sequences in the GenBank database using BLASTN^6^ and were visualized by Easyfig (Sullivan et al., 2011). The closed plasmid was confirmed by PCR (Supplementary Table S4).

## Results

### Phylogenetic analysis of SFP strains and non-SFP strains

**Figure 1:**
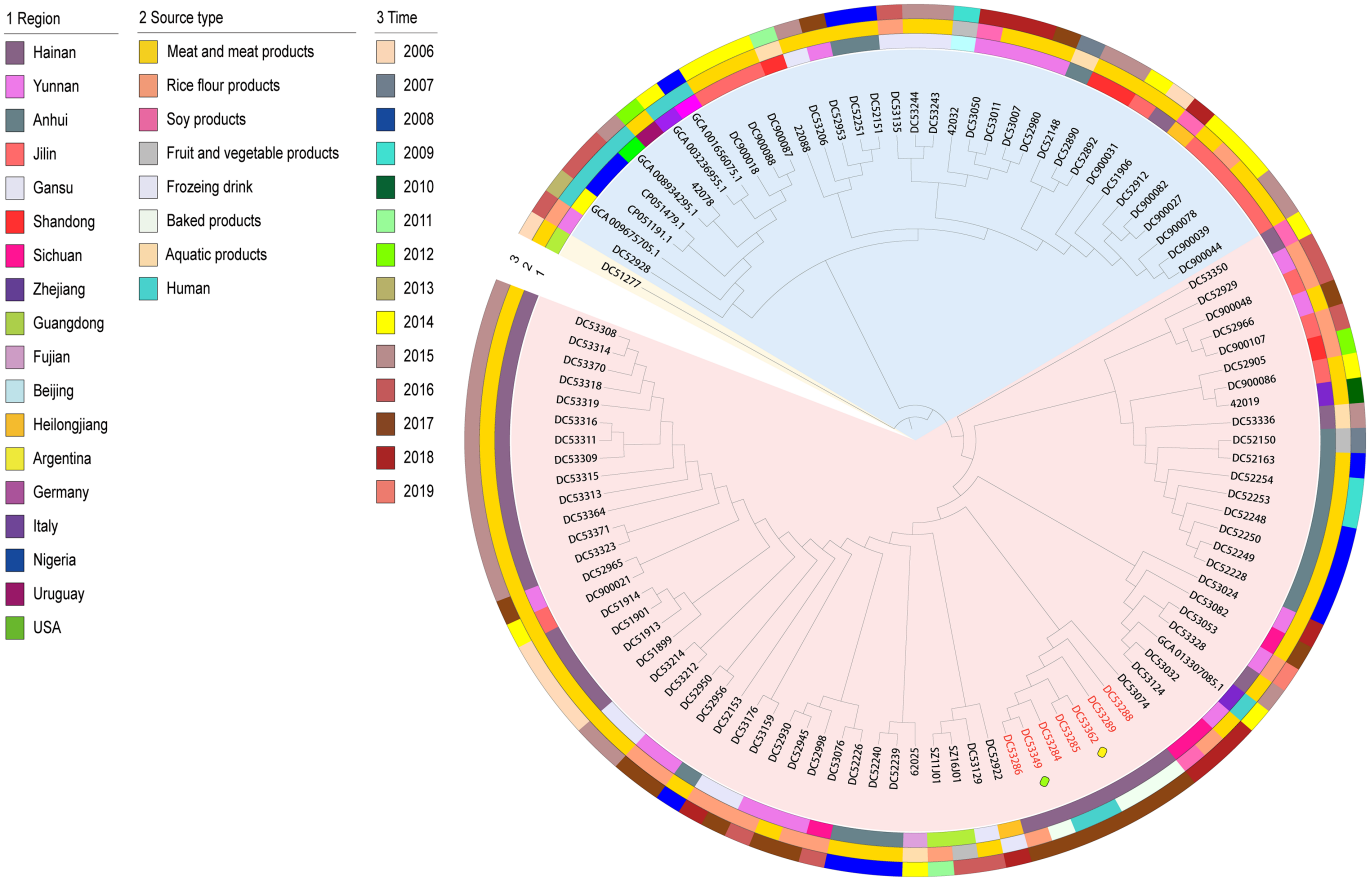
Maximum-likelihood phylogenetic tree based on 2,387 core genes of the 106 *S. aureus* isolates. The outgroup ST943 strain is indicated in yellow bottom; cluster 1 and 2 is shown in red and blue bottom, respectively. Isolates from food poisoning are indicated by red text. The yellow and green boxes represent strains from the cake shop and campus B, respectively. The remaining SFP strains are from campus A. Region, time, and source type are indicated by means of numbers and the colored rings. NCA_013307085.1, GCA_001656075.1, GCA_003236955.1, GCA_008934295.1, GCA_009675705.1, CP051191, and CP051479 are sequences from NCBI.

The 106 genomic sequences (incuding one outgroup sequence) in this study shared 2,387 core genes. The phylogenetic tree can be mainly divided into two clusters (Figure 1). The tree demonstrated that the SFP outbreak strains isolated from the food matrix (DC53286, DC53288, DC53289, DC53349, and DC53362) clustered together with those of the human cases (DC53284 and DC53285) in a single clade carried by one branch in cluster 1. Further analysis of SFP strains in the phylogenetic tree showed that food isolates from campus A were closly clustered with campus B, and the cream from cake shop closly clustered with patient isolates from campus A.

The geographically clustered stains were from Hainan, Anhui, and Jilin Provinces in cluster 1 and 2, respectively. These strains were mainly isolated from meat with adjacent isolation years. The one NCBI sequence from human in Zhejiang Province in China that was closly clustered with Chinese isolates from food matrix in cluster 1. In cluster 2, six NCBI human sequences of foreign countries clustered as a subclade with an imported food isolate from Uruguay and a few food isolates from China.

### Phenotype and genotype of antibiotic resistance

**Figure 2:**
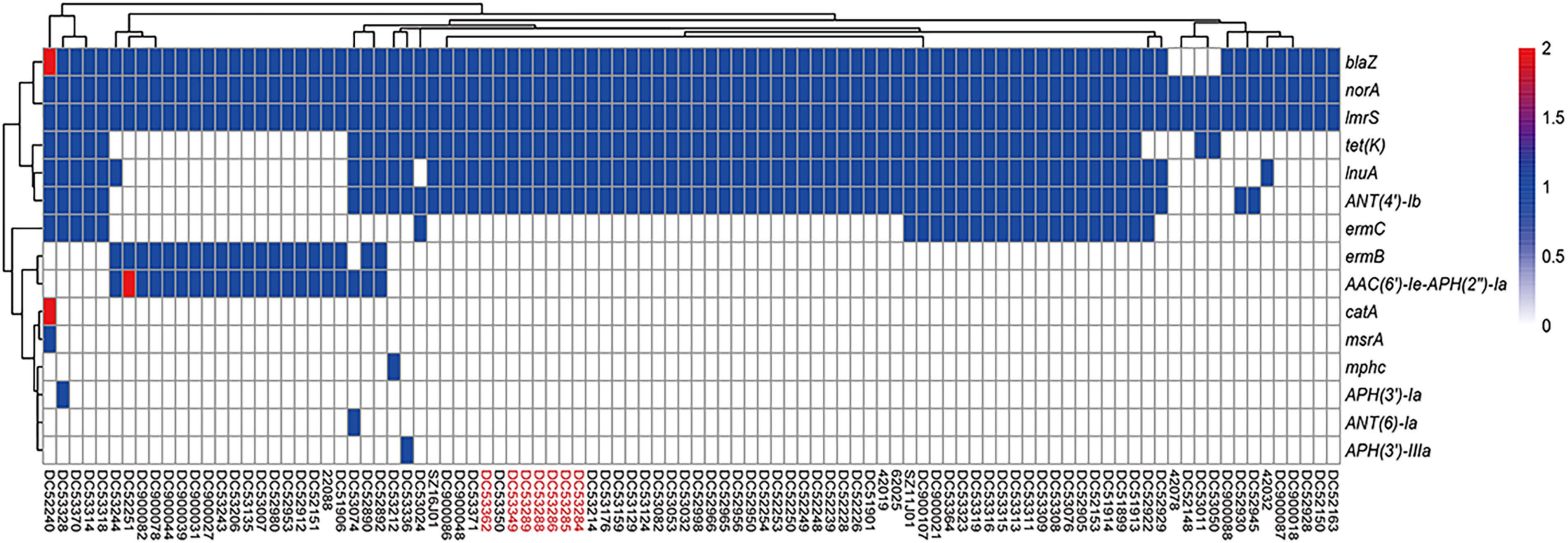
Heatmap of copy number of resistance genes of ST7 *S. aureus* strains. The gene copy number 0, 1, 2 are shown in white, blue, and red bottom, respectively. Isolates from food poisoning are indicated by red text.

All SFP isolates were methicillin-sensitive *S. aureus* (MSSA) and only resistant to penicillin and tetracycline. The heatmap showed the same resistance gene profiles of the seven SFP strains, all of which carried one copy of *blaZ, tetK, ANT (4′)-Ib*, and *lnuA, lmrS*, and *norA* gene (Figure 2).

In this study, 91 food-borne strains carried a total of 15 resistance genes. Six antibiotic genes showed a higher prevalence rate, i.e., *norA, lmrS, blaZ, ANT (4′)-Ib, tetK*, and *lnuA* (Supplementary Table S2). Only two strains carried two copies of resistance genes, which were *AAC (6′)-Ie-APH (2″)-Ia, catA,* and *blaZ*, respectively (Figure 2). No special antibiotic genes were observed for the 7 SFP strains compared with 91 food-borne strains.

Combined with phylogenetic tree, *ermC, lnuA, ANT (4 ‘)-Ib,* and *tetK* were mainly distributed in cluster 1 strains (*p* < 0.05). And most of strains were from poultry and livestock meat in Anhui, Hainan, and Yunnan Province. While *AAC (6 ‘)-Ie-APH (2″)-Ia* and *ermB* genes were mainly found in cluster 2 strains (*p* < 0.05), and most of which were from poultry and livestock meat in Jilin and Gansu Province (Supplementary Table S2; Figure 1).

### Distribution of virulence genes

**Figure 3:**
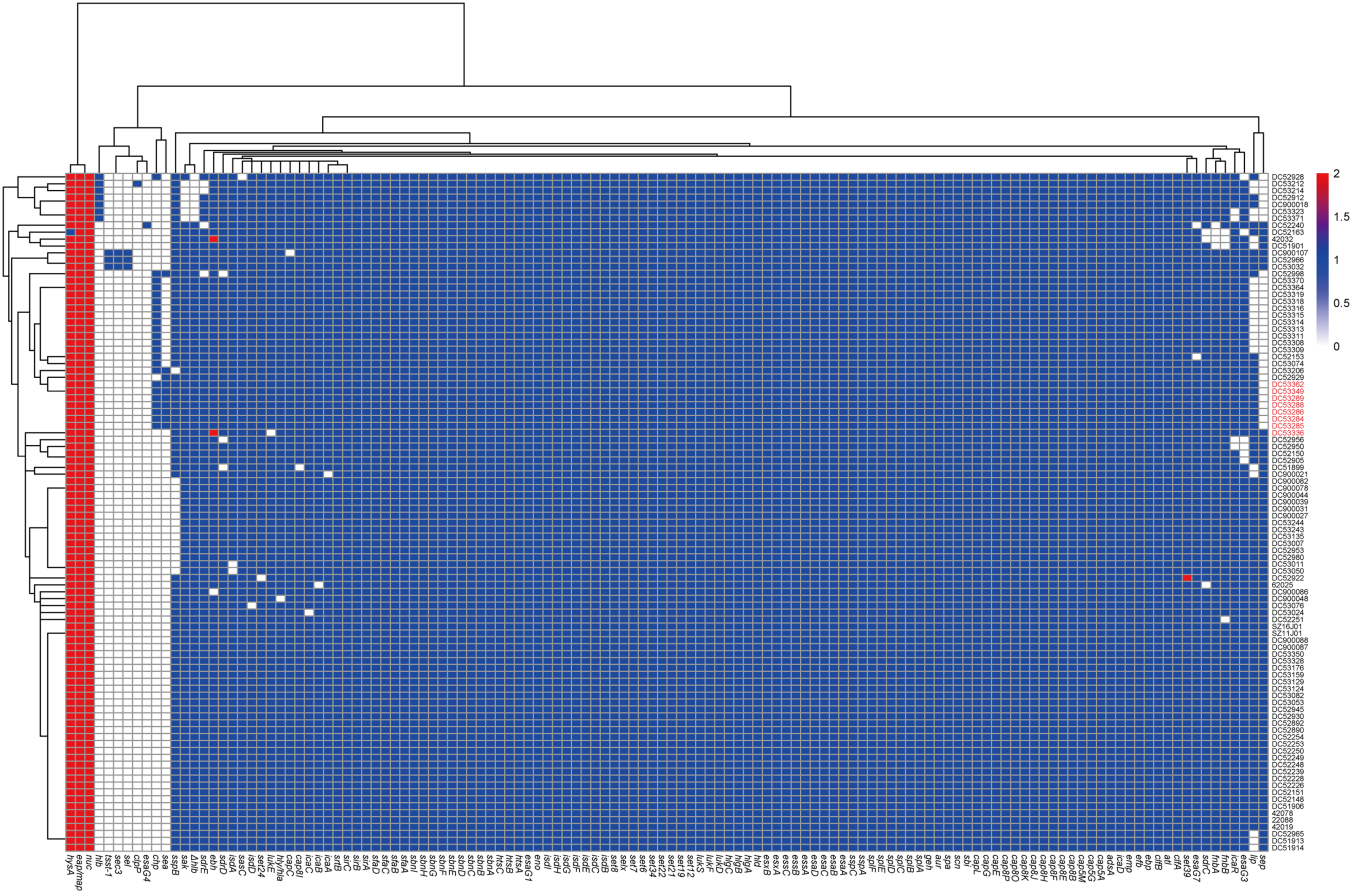
Heatmap of copy number of virulence genes of ST7 *S. aureus* strains. The gene copy number 0, 1, 2 are shown in white, blue, and red bottom, respectively. Isolates from food poisoning are indicated by red text. *Δhlb* is indicated truncated *hlb* gene.

All SFP isolates had the same virulence gene profile (Figure 3; Supplementary Table S3). Among the 27 enterotoxin genes, only *sea* and *selx* were found in all SFP strains. The *chp*, *sak*, *scn*, *lukD, lukE, lukS-PV*, and *lukF-PV* were also present in all SFP strains.

The 91 non-SFP isolates showed a significantly higher prevalence rate of ten virulence genes, i.e., *fnbA, fnbB, sdrD, lukD, lukE, sep, lukF*-PV, *lukS*-PV, *sak*, and *sdrE* compared with the reference data (*p* < 0.05). Six virulence genes (*tsst-1, sea, sec, sel, chp*, and *sspB*) had a lower prevalence rate (*p* < 0.05, Supplementary Table S3). Almost all the virulence genes were one copy, except for *eap/map*, *hysA*, and *nuc.*

### Genomic structure of prophage carrying sea

**Figure 4:**
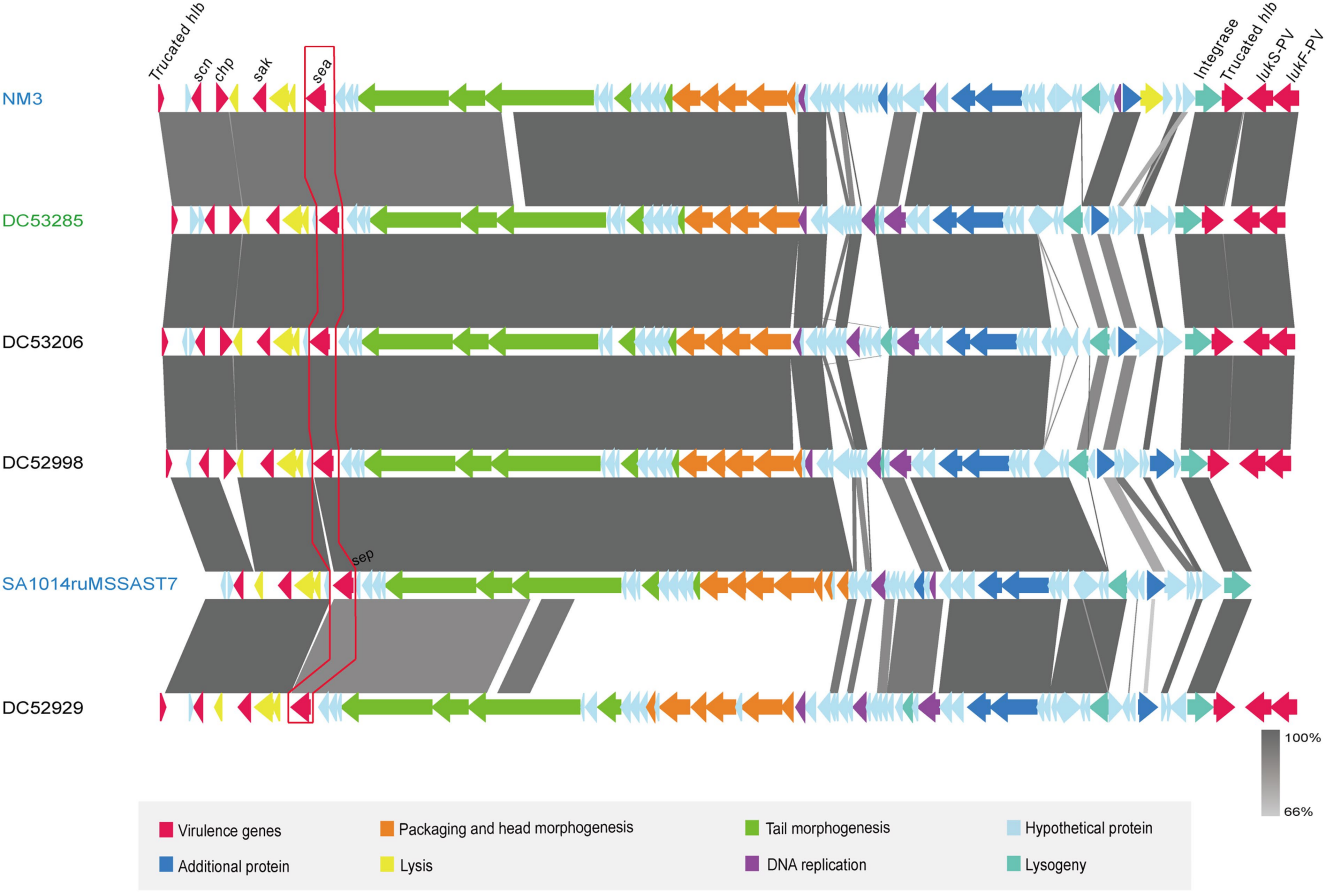
Architecture of prophages from ST7 SFP and non-SFP strains, and similar regions in phage from NM3 and SA1014ruMSSAST7. Corresponding CDSs are colored as indicated. SFP strain DC53285 is shown in green, and non-SFP strains DC53206, DC52998, and DC52929 are shown in black. NM3 (NC_008617.1) and SA1014ruMSSAST7 (NC_048710.1) from NCBI are shown in blue. The nucleotide similarities in the structure are indicated by grey shading.

One SFP strain (DC53285) and three non-SFP strains (DC53206, DC52998, and DC52929) were selected for the prophage structure analysis. The identified prophages of DC53285, DC53206, DC52998, and DC52929 were 42,263 bp, 47,075 bp, 46,733 bp, and 47,230 bp in length and contained 67, 67, 68, and 68 coding sequences (CDSs), respectively. They were all integrated within the *hlb* and belonged to ФSa3int integrase gene group. Overall, the structure of DC53285 shared 98.97% of nucleotide sequence identity and 85% coverage with ФNM3 (NC_008617.1), and shared 96.87% identity and 78% coverage with SA1014ruMSSAST7 (NC_048710.1) (Figure 4). DC52929 prophage showed low nucleotide similarity with the above three prophages. Type A immune evasion cluster (IEC) genes (*sea, sak, chp*, and *scn*) were found in DC53285, DC53206, and DC52998 prophage, while type D IEC genes (*sea, sak, and scn*) were found in DC52929. The *lukF*-PV and *lukS*-PV were located downstream of the four prophages (Figure 4).

### ST7 SFP strain carried a multi-antibiotic resistant plasmid

**Figure 5:**
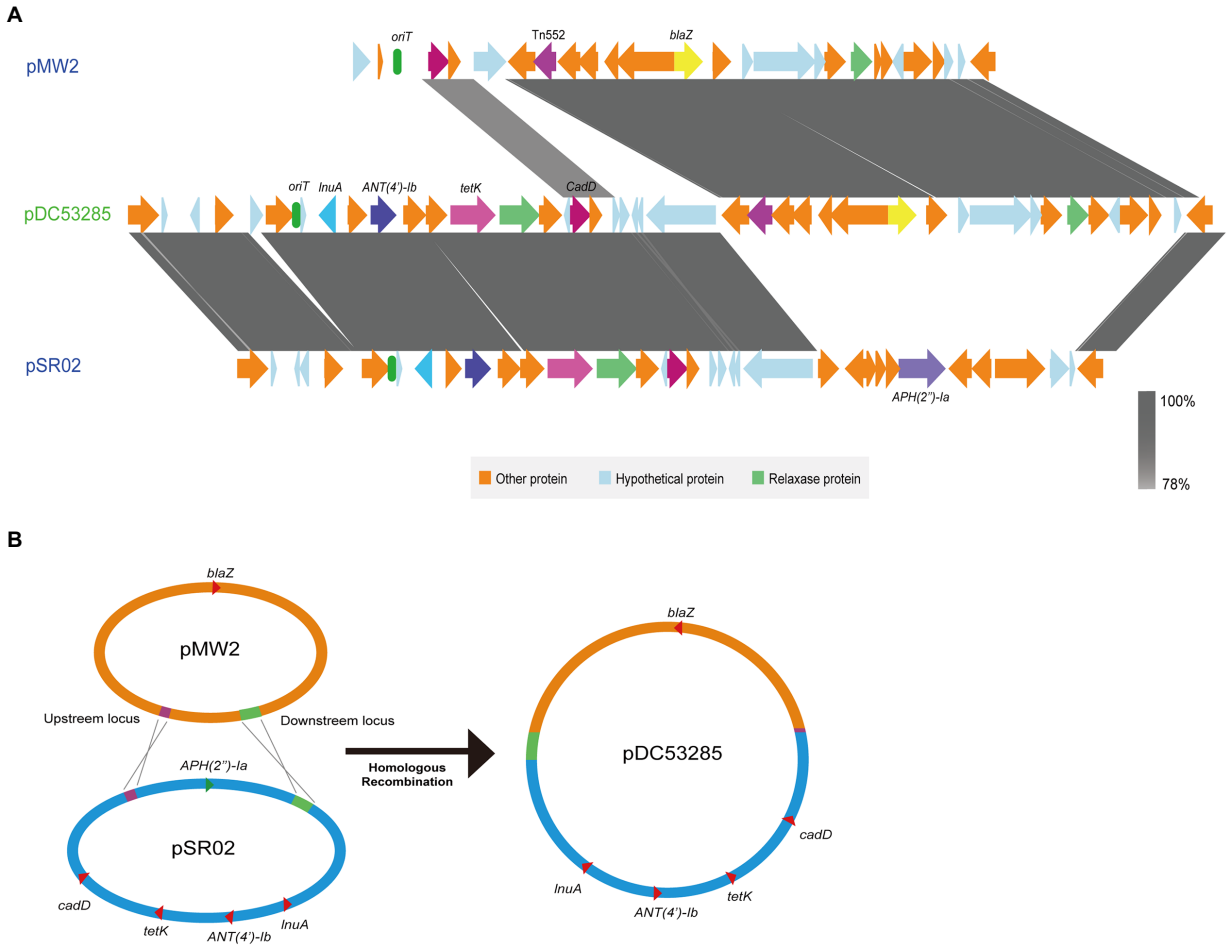
**(A)** Structure of pDC53285 from ST7 SFP strain and similar regions in plasmid pMW2 (AP004832.1) and pSR02(CP048645.1). Corresponding CDSs are colored as indicated. The nucleotide similarities in the structure are indicated by grey shading. **(B)** Illustration of the process of evolution of pMW2 (AP004832.1) and pSR02(CP048645.1) to pDC53285.

A multiple resistance plasmid pDC53285 was present in the SFP strain DC53285. The complete sequence of pDC53285 was 35,024 bp in size and had a G + C content of 29% (accession no. NMDC60045017). Sequence analysis identified 43 CDSs. pDC53285 carried four antibiotic resistance genes, including *ANT(4′)-Ib, tetK, lunA,* and *blaZ*. Plasmid pDC53285 shared 99% coverage (99.97% nucleotide identity) with the plasmid MJ015 (NZ_CP038185.1) from Zhejiang Province in NCBI database. The genetic structure of pDC53285 also showed high nucleotide similarity (71% identity) and the main ORF features with two plasmids pSR02 (NZ_CP048645) and pMW2 (NZ_CM007996). The 1–18,870 bp and 33,717–35,024 bp regions of pDC53285 were highly equivalent to the 1–16,494 bp, and 24,746–26,055 bp regions of plasmid pSR02. The pDC53285 plasmid retained around two-thirds of the genes from pSR02, mainly including *ANT(4′)-Ib, tetK*, *lunA*, *oriT* (the origin of transfer site), and a relaxase protein. The 18,853–25,717 bp region of pDC53285 was highly equivalent to the 2,173–20,100 bp region of pMW2, mainly including *blaZ* and a relaxase protein. The two relaxase protein encoding genes belonged to MOB_V_ family (Figure 5A). Further sequence analysis showed that the two plasmids (pMW2 and pSR02) harbored a 17 bp upstream homologous region and an 835 bp downstream homologous region (Figure 5B).

### ST7 SFP isolates carry intact type I and IV restriction-modification (RM) systems

All SFP strains contained complete type I and type IV RM systems. Analysis of CRISPR-cas system revealed that none of the SFP strains carried CRISPR-cas system encoding genes.

## Discussion

In this study, we used WGS to investigate *S. aureus* involved in the outbreak in Hainan Province, China. We confirmed seven ST7 SFP isolates with a clear phylogenetic clustering in cluster 1. Further analysis of SFP strains in the phylogenetic tree showed that food isolates from campus A were closly clustered with campus B, and the cream from cake shop closly clustered with patient isolates from campus A. Therefore, we concluded that this SFP event was caused by the contamination of cakes from cake shop with *S.aureus* carrying *sea*. The strain isolated from the porridge was also related with this SFP, which may be due to cross contamination of porridge with cakes. SFP investigations have typically relied on epidemiological data and molecular typing; however, WGS is increasingly used in recent years (Mossong et al., 2015; Durand et al., 2018; Nouws et al., 2021). In this study, we also confirmed that WGS can be a high discriminative tool to support epidemiological outbreak-investigations.

SEA was the main cause of food poisoning caused by ST7 *S. aureus* in this study. SEA, alone or in association with other SE(s), is the most frequently involved (>75%) in SFP outbreaks worldwide (Hennekinne et al., 2012; Guillier et al., 2016). The *sea* is the most frequently identified enterotoxin gene in China (Yan et al., 2012; Chen and Xie, 2019) and is always located on the prophage (Argudin et al., 2010), which formed the IEC together with the different combinations of *scn, chp, sak*, and *sep* (van Wamel et al., 2006). All SFP isolates in this study carried ФSa3int prophages belonging to the family *Siphoviridae* and genus *Biseptimavirus.* In addition, type A IEC, including *sea, scn, sak*, and *chp* gene were found on the ФSa3int prophages. Studies have shown that the main difference between human and animal strains of *S. aureus* is that human strains always carry IEC (McCarthy and Lindsay, 2013). Thus, the real source of the contamination of cakes in this study was probably human based on the IEC type of the SFP strains. However, we do not have strains isolated from the staff in the cake shop to support this inference. The prophage of SFP strain DC53285 showed high nucleotide similarity with NM3(NC_008617.1) which was carried by ST254 (CC8) *S. aureus*. Phage ФphiNM3 has been reported in different MLST type strains, including ST1, ST5, ST7, ST8, ST30, ST72, ST109, and ST508 (Nepal et al., 2021). Therefore, the ФSa3int prophage of SFP strains may have been exchanged by horizontal gene transfer (HGT) from the same or different ST strains. A previous study found a significantly higher prevalence of ФNM3 in chronic rhinosinusitis patients with high disease severity compared to those with low disease severity (Nepal et al., 2021). This implies that the prophage of SFP strains can increase the pathogenicity of strains.

A multiple resistant plasmid pDC53285 with *rep5a* was found in ST7 SFP strain. pDC53285 carried *oriT* and relaxase protein, which were similar to that in plasmid pMW2 and pSR02. The conjugative mobilization plasmids usually have a Type-IV secretion apparatus, an origin of transfer (*oriT*) site, and a relaxase protein. Interestingly, it has recently been demonstrated that mobilization transfer can be carried out through the relaxase-*in trans* mechanism for the plasmid only with *oriT* and relaxase protein, and it is a frequent event that most *S. aureus* plasmids have evolved to take advantage of (O'Brien et al., 2015; Ramsay et al., 2016). Therefore, the plasmid pDC53285 in this study may be a mobilizable plasmid through the relaxase-*in trans* mechanism of mobilization. pDC53285 showed the highest nucleotide similarity with the plasmid carried by MJ015 (NZ_CP038185.1), which is an MSSA ST7 strain isolated from a patient in Zhejiang, China. Therefore, pDC53285 may potentially be a mobilizable plasmid through a horizontal transfer manner. Further studies should investigate the distribution of pDC53285 in Chinese strains. Based on the genetic structure of pDC53285, it is speculated that pDC53285 was generated as a result of fusion between plasmids pSR02 and pMW2. On further comparative analysis, two plasmids (pMW2 and pSR02) were found to harbor a 17 bp upstream homologous region and an 835 bp downstream homologous region, facilitating homologous recombination of the two plasmids, and resulting in formation of a 35,024 bp integrated multi-antibiotic resistance plasmid. The same recombination mechanism was reported for a conjugative virulence plasmid, p15WZ-82_Vir, from a clinical *Klebsiella variicola* strain (Yang et al., 2020).

*ANT(4′)-Ib*, also called *aadD2*, encoding an aminoglycoside 4′-O-nucleotidyltransferase was first described in *Bacillus clausii* and rarelly reported in *S. aureus*(Bozdogan et al., 2003; Ullah et al., 2021). In this study, the high prevalence of *ANT (4′)-Ib,* together with *blaZ, tetK,* and *lnuA* were found in ST7 food-borne and SFP strains. It may be that these food-borne strains carry the similar multiple resistant plasmid as SFP strains as well. These antibiotic resistance genes were mainly distributed in strains of cluster 1 and closely associated with region and poutry/livestorck-derived foods. Whether these resistance genes originated in local poutry/livestorck associated strains is worth to be further investigated.

The *sep* gene is always located on the prophage and can also cause SFP (Omoe et al., 2005; Chiang et al., 2008; Argudin et al., 2010). The carriage rate of *sep* carried by ST7 *S. aureus* in this study (69.4%, 68/98) was much higher than that in previous studies (Omoe et al., 2005; Bania et al., 2006; Chiang et al., 2008; Wang et al., 2021). Therefore, the ST7 strains isolated from food in this study are at high risk of causing SFP. This is the first study to identify a high carriage rate of *lukFS*-PV (100%, 98/98) in the ST7 *S. aureus*. The *lukFS*-PV plays an important role in pathogenicity in the context of bacteremia, keratitis, skin and soft tissue infections, and other related diseases (Sueke et al., 2013; Knudsen et al., 2016; Xiao et al., 2019). Such a high carriage rate of *lukFS*-PV among ST7 isolates is liable to increase the risk and severity of diseases.

In summary, we concluded that this SFP event was caused by the contamination of cakes from cake shop with ST7 *S. aureus*. The food poisoning was found to be caused by enterotoxin A and a type A IEC (*sea, scn, chp, and sak*) was located on the ФSa3int prophage. Six antibiotic genes including *blaZ, ANT (4′)-Ib, tetK, lnuA, norA,* and *lmrS* were present in all SFP strains and also showed a higher prevalence rate in 91 food-borne strains. In addition, a multi-resistant plasmid pDC53285 was found in ST7 SFP strains, which may act as a vector for the dissemination of antimicrobial resistance among strains. Due attention should be accorded to the high prevalence rate of *sep, lukF*-PV, and *lukS*-PV in ST7 strains isolated from food, which is liable to increase the risk of SFP and other diseases.

## Limitations

Because this is a retrospective study, samples from the staff in the cake shop, food handlers, and environment in the canteen in the kindergarten were not available. It is difficult to conclude whether the contamination comes from human.

## Data availability statement

‘The data presented in the study are deposited in the NCBI repository, accession number PRJNA917243, and modified BioSample Accession is available in the Supplementary material.

## Ethics statement

This study was approved by Ethical Committee of National Institute for Communicable Disease Control and Prevention Chinese Center for Disease Control and Prevention (approval No. ICDC-202111). The patients/participants provided written informed consent to participate in this study.

## Author contributions

All authors listed have made a substantial, direct, and intellectual contribution to the work and approved it for publication.

## Funding

This work was supported by the National Natural Science Foundation of China (Grant No. 81873959), Key R&D Program of China (2018YFC1603800) and The National Key R&D Program of China (2021YFC2301000).

## Conflict of interest

The authors declare that the research was conducted in the absence of any commercial or financial relationships that could be construed as a potential conflict of interest.

## Publisher’s note

All claims expressed in this article are solely those of the authors and do not necessarily represent those of their affiliated organizations, or those of the publisher, the editors and the reviewers. Any product that may be evaluated in this article, or claim that may be made by its manufacturer, is not guaranteed or endorsed by the publisher.

## Footnotes

^1^ https://itol.embl.de

^2^ https://pubmlst.org/

^3^ https://cge.cbs.dtu.dk/services/Restriction-ModificationFinder/

^4^ http://phaster.ca/

^5^ https://cge.food.dtu.dk/services/PlasmidFinder/

^6^ http://blast.ncbi.nlm.nih.gov/blast

## References

[ref1] Argudin M. A. Mendoza M. C. Rodicio M. R. (2010). Food poisoning and *aureus* enterotoxins. Toxins (Basel) 2, 1751–1773. doi: 10.3390/toxins2071751, PMID: 22069659 PMC3153270

[ref2] Bania J. Dabrowska A. Bystron J. Korzekwa K. Chrzanowska J. Molenda J. (2006). Distribution of newly described enterotoxin-like genes in *Staphylococcus aureus* from food. Int. J. Food Microbiol. 108, 36–41. doi: 10.1016/j.ijfoodmicro.2005.10.013, PMID: 16380185

[ref3] Bankevich A. Nurk S. Antipov D. Gurevich A. A. Dvorkin M. Kulikov A. S.. (2012). SPAdes: a new genome assembly algorithm and its applications to single-cell sequencing. J. Comput. Biol. 19, 455–477. doi: 10.1089/cmb.2012.0021, PMID: 22506599 PMC3342519

[ref4] Bastos C. P. Bassani M. T. Mata M. M. Lopes G. V. da Silva W. P. (2017). Prevalence and expression of staphylococcal enterotoxin genes in *Staphylococcus aureus* isolated from food poisoning outbreaks. Can. J. Microbiol. 63, 834–840. doi: 10.1139/cjm-2017-0316, PMID: 28820948

[ref5] Bozdogan B. Galopin S. Gerbaud G. Courvalin P. Leclercq R. (2003). Chromosomal aadD2 encodes an aminoglycoside nucleotidyltransferase in *Bacillus clausii*. Antimicrob. Agents Chemother. 47, 1343–1346. doi: 10.1128/AAC.47.4.1343-1346.2003, PMID: 12654668 PMC152513

[ref6] Cha J. O. Lee J. K. Jung Y. H. Yoo J. I. Park Y. K. Kim B. S.. (2006). Molecular analysis of *Staphylococcus aureus* isolates associated with staphylococcal food poisoning in South Korea. J. Appl. Microbiol. 101, 864–871. doi: 10.1111/j.1365-2672.2006.02957.x, PMID: 16968298

[ref7] Chan B. K. C. (2018). Data Analysis Using R Programming. Adv. Exp. Med. Biol. 1082, 47–122. doi: 10.1007/978-3-319-93791-5_2 30357717

[ref8] Chen Q. Xie S. (2019). Genotypes, enterotoxin gene profiles, and antimicrobial resistance of *Staphylococcus aureus* associated with foodborne outbreaks in Hangzhou, China. Toxins (Basel) 11:307. doi: 10.3390/toxins11060307, PMID: 31146460 PMC6628443

[ref9] Chen S. Zhou Y. Chen Y. Gu J. (2018). Fastp: an ultra-fast all-in-one FASTQ preprocessor. Bioinformatics 34, i884–i890. doi: 10.1093/bioinformatics/bty560, PMID: 30423086 PMC6129281

[ref10] Chiang Y. C. Liao W. W. Fan C. M. Pai W. Y. Chiou C. S. Tsen H. Y. (2008). PCR detection of staphylococcal enterotoxins (SEs) N, O, P, Q, R, U, and survey of SE types in *Staphylococcus aureus* isolates from food-poisoning cases in Taiwan. Int. J. Food Microbiol. 121, 66–73. doi: 10.1016/j.ijfoodmicro.2007.10.005, PMID: 18068843

[ref11] Couvin D. Bernheim A. Toffano-Nioche C. Touchon M. Michalik J. Néron B.. (2018). CRISPRCasFinder, an update of CRISRFinder, includes a portable version, enhanced performance and integrates search for Cas proteins. Nucleic Acids Res. 46, W246–W251. doi: 10.1093/nar/gky425, PMID: 29790974 PMC6030898

[ref12] Durand G. Javerliat F. Bes M. Veyrieras J. B. Guigon G. Mugnier N.. (2018). Routine whole-genome sequencing for outbreak investigations of *Staphylococcus aureus* in a National Reference Center. Front. Microbiol. 9:511. doi: 10.3389/fmicb.2018.00511, PMID: 29616014 PMC5869177

[ref13] Gu F. F. Chen Y. Dong D. P. Song Z. Guo X. K. Ni Y. X.. (2016). Molecular epidemiology of *Staphylococcus aureus* among patients with skin and soft tissue infections in two Chinese hospitals. Chin. Med. J. 129, 2319–2324. doi: 10.4103/0366-6999.190673, PMID: 27647191 PMC5040018

[ref14] Guillier L. Bergis H. Guillier F. Noel V. Auvray F. Hennekinne J.-A. (2016). Dose-response modelling of staphylococcal enterotoxins using outbreak data. Procedia Food Sci. 7, 129–132. doi: 10.1016/j.profoo.2016.05.002

[ref15] Hennekinne J. A. De Buyser M. L. Dragacci S. (2012). *Staphylococcus aureus* and its food poisoning toxins: characterization and outbreak investigation. FEMS Microbiol. Rev. 36, 815–836. doi: 10.1111/j.1574-6976.2011.00311.x, PMID: 22091892

[ref16] Knudsen T. A. Skov R. Petersen A. Larsen A. R. Benfield T. Danish Staphylococcal Bacteremia Study (2016). Increased age-dependent risk of death associated with lukF-PV-positive *Staphylococcus aureus* bacteremia. Open Forum Infect. Dis. 3:ofw220. doi: 10.1093/ofid/ofw220, PMID: 27957504 PMC5146761

[ref17] Letunic I. Bork P. (2019). Interactive tree of life (iTOL) v4: recent updates and new developments. Nucleic Acids Res. 47, W256–W259. doi: 10.1093/nar/gkz239, PMID: 30931475 PMC6602468

[ref18] Li H. Li W. Dai Y. Jiang Y. Liang J.. (2021). Characteristics of settings and etiologic agents of foodborne disease outbreaks–China, 2020. China CDC Wkly 3, 889–893. doi: 10.46234/ccdcw2021.219, PMID: 34733577 PMC8545604

[ref19] Li T. M. Lu H. Y. Wang X. Gao Q. Q. Dai Y. X. Shang J.. (2017). Molecular characteristics of *Staphylococcus aureus* causing bovine mastitis between 2014 and 2015. Front. Cell. Infect. Microbiol. 7:127. doi: 10.3389/fcimb.2017.00127, PMID: 28469994 PMC5395632

[ref20] Li S. G. Sun S. J. Yang C. T. Chen H. B. Yin Y. Y. Li H. N.. (2018). The changing pattern of population structure of *Staphylococcus aureus* from bacteremia in China from 2013 to 2016: ST239-030-MRSA replaced by ST59-t437. Front. Microbiol. 9:332. doi: 10.3389/fmicb.2018.00332 29535697 PMC5835333

[ref21] Liao F. Gu W. P. Yang Z. S. Mo Z. S. Fan L. Guo Y. D.. (2018). Molecular characteristics of *Staphylococcus aureus* isolates from food surveillance in Southwest China. BMC Microbiol. 18:91. doi: 10.1186/s12866-018-1239-z, PMID: 30157758 PMC6114054

[ref22] Liu J. K. Bai L. Li W. W. Han H. H. Fu P. Ma X. C.. (2018). Trends of foodborne diseases in China: lessons from laboratory-based surveillance since 2011. Front. Med. 12, 48–57. doi: 10.1007/s11684-017-0608-6, PMID: 29282610

[ref23] Lv G. Jiang R. Zhang H. Wang L. Li L. Gao W.. (2021). Molecular characteristics of *Staphylococcus aureus* from food samples and food poisoning outbreaks in Shijiazhuang, China. Front. Microbiol. 12:652276. doi: 10.3389/fmicb.2021.652276, PMID: 34239506 PMC8258372

[ref24] McCarthy A. J. Lindsay J. A. (2013). *Staphylococcus aureus* innate immune evasion is lineage-specific: a bioinfomatics study. Infect. Genet. Evol. 19, 7–14. doi: 10.1016/j.meegid.2013.06.012, PMID: 23792184

[ref25] Merlino J. Watson J. Rose B. Beard-Pegler M. Gottlieb T. Bradbury R.. (2002). Detection and expression of methicillin/oxacillin resistance in multidrug-resistant and non-multidrug-resistant *Staphylococcus aureus* in Central Sydney, Australia. J. Antimicrob Chemother. 49, 793–801. doi: 10.1093/jac/dkf021, PMID: 12003973

[ref26] Mossong J. Decruyenaere F. Moris G. Ragimbeau C. Olinger C. M. Johler S.. (2015). Investigation of a staphylococcal food poisoning outbreak combining case-control, traditional typing and whole genome sequencing methods, Luxembourg, June 2014. Euro Surveill. 20:30059. doi: 10.2807/1560-7917.ES.2015.20.45.30059, PMID: 26608881

[ref27] Nepal R. Houtak G. Shaghayegh G. Bouras G. Shearwin K. Psaltis A. J.. (2021). Prophages encoding human immune evasion cluster genes are enriched in *Staphylococcus aureus* isolated from chronic rhinosinusitis patients with nasal polyps. Microb Genom 7:726. doi: 10.1099/mgen.0.000726, PMID: 34907894 PMC8767322

[ref28] Nouws S. Bogaerts B. Verhaegen B. Denayer S. Laeremans L. Marchal K.. (2021). Whole genome sequencing provides an added value to the investigation of staphylococcal food poisoning outbreaks. Front. Microbiol. 12:750278. doi: 10.3389/fmicb.2021.750278, PMID: 34795649 PMC8593433

[ref29] O'Brien F. G. Yui Eto K. Murphy R. J. Fairhurst H. M. Coombs G. W. Grubb W. B.. (2015). Origin-of-transfer sequences facilitate mobilisation of non-conjugative antimicrobial-resistance plasmids in *Staphylococcus aureus*. Nucleic Acids Res. 43, 7971–7983. doi: 10.1093/nar/gkv755, PMID: 26243776 PMC4652767

[ref30] Omoe K. Hu D. L. Takahashi-Omoe H. Nakane A. Shinagawa K. (2005). Comprehensive analysis of classical and newly described staphylococcal superantigenic toxin genes in *Staphylococcus aureus* isolates. FEMS Microbiol. Lett. 246, 191–198. doi: 10.1016/j.femsle.2005.04.007, PMID: 15899405

[ref31] Page A. J. Cummins C. A. Hunt M. Wong V. K. Reuter S. Holden M. T.. (2015). Roary: rapid large-scale prokaryote pan genome analysis. Bioinformatics 31, 3691–3693. doi: 10.1093/bioinformatics/btv421, PMID: 26198102 PMC4817141

[ref32] Pinchuk I. V. Beswick E. J. Reyes V. E. (2010). Staphylococcal enterotoxins. Toxins (Basel) 2, 2177–2197. doi: 10.3390/toxins2082177, PMID: 22069679 PMC3153290

[ref33] Ramsay J. P. Kwong S. M. Murphy R. J. Yui Eto K. Price K. J. Nguyen Q. T.. (2016). An updated view of plasmid conjugation and mobilization in staphylococcus. Mob. Genet. Elements 6:e1208317. doi: 10.1080/2159256X.2016.1208317, PMID: 27583185 PMC4993578

[ref34] Rong D. L. Wu Q. P. Wu S. Zhang J. M. Xu M. F. (2018). Distribution and drug resistance and genotyping of *Staphylococcus aureus* contamination in ready-to-eat food and vegetables in some parts of China. Acta Microbiol Sin. 58, 314–323. doi: 10.13343/j.cnki.wsxb.20170144

[ref35] Seemann T. (2014). Prokka: rapid prokaryotic genome annotation. Bioinformatics 30, 2068–2069. doi: 10.1093/bioinformatics/btu153, PMID: 24642063

[ref36] Slingerland B. Vos M. C. Bras W. Kornelisse R. F. De Coninck D. van Belkum A.. (2020). Whole-genome sequencing to explore nosocomial transmission and virulence in neonatal methicillin-susceptible *Staphylococcus aureus* bacteremia. Antimicrob. Resist. Infect. Control 9:39. doi: 10.1186/s13756-020-0699-8, PMID: 32087747 PMC7036242

[ref37] Stamatakis A. (2014). RAxML version 8: a tool for phylogenetic analysis and post-analysis of large phylogenies. Bioinformatics 30, 1312–1313. doi: 10.1093/bioinformatics/btu033, PMID: 24451623 PMC3998144

[ref38] Sueke H. Shankar J. Neal T. Winstanley C. Tuft S. Coates R.. (2013). lukSF-PV in *Staphylococcus aureus* keratitis isolates and association with clinical outcome. Invest. Ophthalmol. Vis. Sci. 54, 3410–3416. doi: 10.1167/iovs.12-11276 23580488

[ref39] Sullivan M. J. Petty N. K. Beatson S. A. (2011). Easyfig: a genome comparison visualizer. Bioinformatics 27, 1009–1010. doi: 10.1093/bioinformatics/btr039, PMID: 21278367 PMC3065679

[ref40] Suzuki Y. Omoe K. Hu D. L. Sato'o Y. Ono H. K. Monma C.. (2014). Molecular epidemiological characterization of *Staphylococcus aureus* isolates originating from food poisoning outbreaks that occurred in Tokyo, Japan. Microbiol. Immunol. 58, 570–580. doi: 10.1111/1348-0421.12188, PMID: 25088705

[ref001] Ullah N. Dar H. A. Naz K. Andleeb S. Rahman A. Saeed M. T.. (2021). Genomic Investigation of Methicillin-Resistant Staphylococcus aureus ST113 Strains Isolated from Tertiary Care Hospitals in Pakistan. Antibiotics (Basel). 10:1121. doi: 10.3390/antibiotics10091121 34572703 PMC8465543

[ref41] van Wamel W. J. Rooijakkers S. H. Ruyken M. van Kessel K. P. van Strijp J. A. (2006). The innate immune modulators staphylococcal complement inhibitor and chemotaxis inhibitory protein of *Staphylococcus aureus* are located on beta-hemolysin-converting bacteriophages. J. Bacteriol. 188, 1310–1315. doi: 10.1128/JB.188.4.1310-1315.2006, PMID: 16452413 PMC1367213

[ref42] Wang W. Baker M. Hu Y. Xu J. Yang D. Maciel-Guerra A.. (2021). Whole-genome sequencing and machine learning analysis of *Staphylococcus aureus* from multiple heterogeneous sources in China Reveals Common Genetic Traits of Antimicrobial Resistance. mSystems 6:e0118520. doi: 10.1128/mSystems.01185-20, PMID: 34100643 PMC8579812

[ref43] Wick R. R. Judd L. M. Gorrie C. L. Holt K. E. (2017). Unicycler: resolving bacterial genome assemblies from short and long sequencing reads. PLoS Comput. Biol. 13:e1005595. doi: 10.1371/journal.pcbi.1005595, PMID: 28594827 PMC5481147

[ref44] Wu S. Huang J. Wu Q. Zhang J. Zhang F. Yang X.. (2018). *Staphylococcus aureus* isolated from retail meat and meat products in China: incidence, antibiotic resistance and genetic diversity. Front. Microbiol. 9:2767. doi: 10.3389/fmicb.2018.02767, PMID: 30498486 PMC6249422

[ref45] Xiao N. Yang J. Duan N. Lu B. Wang L. (2019). Community-associated *Staphylococcus aureus* PVL(+) ST22 predominates in skin and soft tissue infections in Beijing, China. Infect. Drug Resist. 12, 2495–2503. doi: 10.2147/IDR.S212358, PMID: 31616166 PMC6698600

[ref46] Yan X. Song Y. Yu X. Tao X. Yan J. Luo F.. (2015). Factors associated with *Staphylococcus aureus* nasal carriage among healthy people in northern China. Clin. Microbiol. Infect. 21, 157–162. doi: 10.1016/j.cmi.2014.08.023, PMID: 25658548

[ref47] Yan X. Wang B. Tao X. Hu Q. Cui Z. Zhang J.. (2012). Characterization of *Staphylococcus aureus* strains associated with food poisoning in Shenzhen, China. Appl. Environ. Microbiol. 78, 6637–6642. doi: 10.1128/AEM.01165-12, PMID: 22798367 PMC3426705

[ref48] Yan X. Yu X. Tao X. Zhang J. Zhang B. Dong R.. (2014). *Staphylococcus aureus* ST398 from slaughter pigs in Northeast China. Int. J. Med. Microbiol. 304, 379–383. doi: 10.1016/j.ijmm.2013.12.003, PMID: 24418357

[ref49] Yang X. M. Ye L. W. Chan E. W. C. Zhang R. Chen S. (2020). Tracking recombination events that occur in conjugative virulence plasmid p15WZ-82_Vir during the transmission process. mSystems 5:20. doi: 10.1128/mSystems.00140-20 PMC7363002 32665326

[ref50] Ye X. Liu W. Fan Y. Wang X. Zhou J. Yao Z.. (2015). Frequency-risk and duration-risk relations between occupational livestock contact and methicillin-resistant *Staphylococcus aureus* carriage among workers in Guangdong, China. Am. J. Infect. Control 43, 676–681. doi: 10.1016/j.ajic.2015.03.026, PMID: 25934060

[ref51] You Y. Davies M. R. Protani M. McIntyre L. Walker M. J. Zhang J. (2018). Scarlet fever epidemic in China caused by streptococcus pyogenes serotype M12: Epidemiologic and molecular analysis. EBioMedicine 28, 128–135. doi: 10.1016/j.ebiom.2018.01.010 29342444 PMC5835554

[ref52] Zhou Y. He Y. Y. Wang F. W. He P. Hou S. P. Tao X.. (2022). Molecular characterization of *Staphylococcus aureus* ST6 and ST7 isolates from food-borne illness outbreaks. Zhonghua Yu Fang Yi Xue Za Zhi 56, 178–184. doi: 10.3760/cma.j.cn112150-20210712-00670, PMID: 35184447

[ref53] Zhou Y. Li X. Yan H. (2020). Genotypic characteristics and correlation of epidemiology of *Staphylococcus aureus* in healthy pigs, diseased pigs, and environment. Antibiotics (Basel) 9:839. doi: 10.3390/antibiotics9120839, PMID: 33255159 PMC7760503

