# Supplementary figures and images for: A food poisoning caused by ST7 Staphylococcal aureus harboring sea gene in Hainan province, China
Source: Front Microbiol. 2023 Mar 16;14:1110720. doi: 10.3389/fmicb.2023.1110720 (PMC10060626; doi:10.3389/fmicb.2023.1110720)

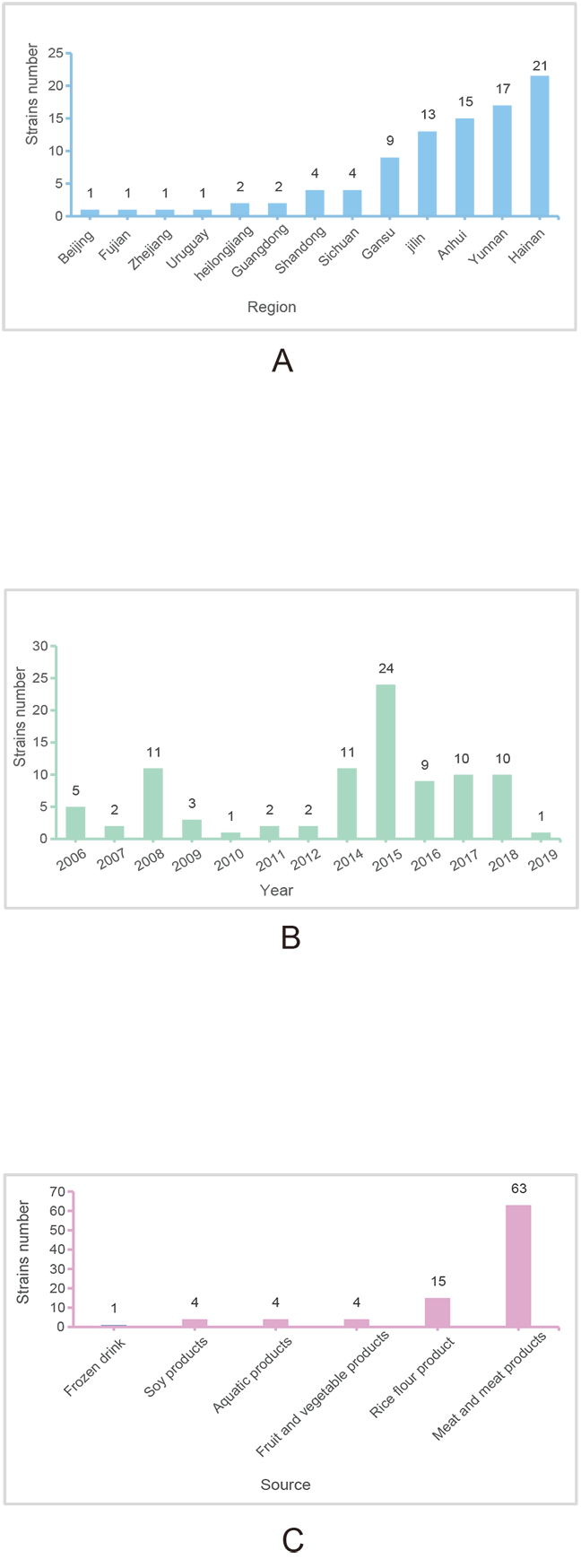

Supplement: Supplementary file 1 [file Image_1.TIF]
