# Supplementary material for: A food poisoning caused by ST7 Staphylococcal aureus harboring sea gene in Hainan province, China
Source: Front Microbiol. 2023 Mar 16;14:1110720. doi: 10.3389/fmicb.2023.1110720 (PMC10060626; doi:10.3389/fmicb.2023.1110720)
Supplement: Supplementary file 2 [file Table_1.docx]

| Basic information of the strains and plasmids in this study | | | | | | | | | | | | | | | |
| --- | --- | --- | --- | --- | --- | --- | --- | --- | --- | --- | --- | --- | --- | --- | --- |
| Isolate name | BioSample Accession | Collection date | Country | Province | Source type | MSSA/MRSA | MLST | Clonal Complex(CC) | Total length of genome | N50 | N90 | Mean length of genome | Max length of genome | Min length of genome | Contig of genome |
| 22088 | SAMN32531304 | 2011/9/19 | China | Shandong | Aquatic product | MSSA | 7 | CC7 | 2788692 | 653829 | 76112 | 99596 | 767152 | 560 | 28 |
| 42019 | SAMN32531305 | 2010/9/23 | China | Zhejiang | Meat product | MSSA | 7 | CC7 | 2796404 | 319713 | 170673 | 155355 | 917860 | 502 | 18 |
| 42032 | SAMN32531306 | 2009/7/13 | China | Beijing | Vegetable product | MSSA | 7 | CC7 | 2715539 | 350763 | 113974 | 150863 | 1018941 | 524 | 18 |
| 42078 | SAMN32531307 | 2012/5/18 | [Uruguay](https://www.baidu.com/link?url=fGuw2RlVSJVYpyyFp5nSqVWTbDK3f4m0qKrPWEtrQAAChmrVu5eKrDlit_ccAwpxpGA2KJ0SEg6Q-YUViyuhnlArNsqDeIQubM-7NwpuYma&wd=&eqid=845c9853000a0aaf00000002622b2948) | NA | Meat | MSSA | 7 | CC7 | 2757234 | 359082 | 170682 | 137861 | 745371 | 528 | 20 |
| 62025 | SAMN32531308 | 2014/4/10 | China | Fujian | Aquatic product | MSSA | 7 | CC7 | 2763201 | 925715 | 109836 | 131581 | 1027046 | 509 | 21 |
| DC51899 | SAMN32531309 | 2006/8/7 | China | Hainan | Meat | MSSA | 7 | CC7 | 2794907 | 653070 | 90747 | 87340 | 769790 | 610 | 32 |
| DC51901 | SAMN32531310 | 2006/8/7 | China | Hainan | Meat | MSSA | 7 | CC7 | 2788945 | 652836 | 90739 | 96170 | 769882 | 610 | 29 |
| DC51906 | SAMN32531311 | 2006/8/7 | China | Hainan | Meat | MSSA | 7 | CC7 | 2747031 | 707358 | 170798 | 171689 | 1362490 | 610 | 16 |
| DC51913 | SAMN32531312 | 2006/8/7 | China | Hainan | Meat | MSSA | 7 | CC7 | 2788781 | 652768 | 96686 | 103288 | 769676 | 590 | 27 |
| DC51914 | SAMN32531313 | 2006/8/7 | China | Hainan | Meat | MSSA | 7 | CC7 | 2788981 | 652769 | 94331 | 99606 | 769678 | 590 | 28 |
| DC52148 | SAMN32531314 | 2007/3/15 | China | Anhui | Aquatic product | MSSA | 7 | CC7 | 2711160 | 1360947 | 170794 | 246469 | 1360947 | 610 | 11 |
| DC52150 | SAMN32531315 | 2007/3/27 | China | Anhui | Vegetable product | MSSA | 7 | CC7 | 2824183 | 706696 | 109888 | 148641 | 1065595 | 529 | 19 |
| DC52151 | SAMN32531316 | 2008/8/3 | China | Anhui | Meat | MSSA | 7 | CC7 | 2797073 | 847391 | 111019 | 199790 | 1027967 | 610 | 14 |
| DC52153 | SAMN32531317 | 2008/8/3 | China | Anhui | Meat | MSSA | 7 | CC7 | 2835462 | 374230 | 171025 | 123280 | 707497 | 576 | 23 |
| DC52163 | SAMN32531318 | 2008/11/2 | China | Anhui | Meat product | MSSA | 7 | CC7 | 2803100 | 1450180 | 51274 | 57206 | 1450180 | 506 | 49 |
| DC52226 | SAMN32531319 | 2008/6/15 | China | Anhui | Meat | MSSA | 7 | CC7 | 2788393 | 556674 | 111751 | 126745 | 847826 | 544 | 22 |
| DC52228 | SAMN32531320 | 2008/8/3 | China | Anhui | Meat | MSSA | 7 | CC7 | 2789489 | 1407751 | 170705 | 199249 | 1407751 | 590 | 14 |
| DC52239 | SAMN32531321 | 2008/8/3 | China | Anhui | Meat | MSSA | 7 | CC7 | 2764551 | 707251 | 170857 | 172784 | 1365019 | 610 | 16 |
| DC52240 | SAMN32531322 | 2008/8/3 | China | Anhui | Meat | MSSA | 7 | CC7 | 2809594 | 2069850 | 170787 | 87799 | 2069850 | 567 | 32 |
| DC52248 | SAMN32531323 | 2008/8/3 | China | Anhui | Meat | MSSA | 7 | CC7 | 2789803 | 1407751 | 170705 | 199271 | 1407751 | 590 | 14 |
| DC52249 | SAMN32531324 | 2008/8/3 | China | Anhui | Meat | MSSA | 7 | CC7 | 2788711 | 1407751 | 170705 | 199193 | 1407751 | 590 | 14 |
| DC52250 | SAMN32531325 | 2008/8/3 | China | Anhui | Meat | MSSA | 7 | CC7 | 2746450 | 705474 | 170707 | 196175 | 1364409 | 590 | 14 |
| DC52251 | SAMN32531326 | 2008/8/3 | China | Anhui | Meat | MSSA | 7 | CC7 | 2796124 | 847473 | 111019 | 174757 | 1027810 | 610 | 16 |
| DC52253 | SAMN32531327 | 2009/6/3 | China | Anhui | Meat | MSSA | 7 | CC7 | 2745114 | 705474 | 170705 | 196079 | 1364470 | 590 | 14 |
| DC52254 | SAMN32531328 | 2009/6/3 | China | Anhui | Meat | MSSA | 7 | CC7 | 2748600 | 705371 | 170667 | 196328 | 1364181 | 728 | 14 |
| DC52890 | SAMN32531329 | 2015 | China | Shandong | Meat | MSSA | 7 | CC7 | 2806284 | 767288 | 170964 | 155904 | 815284 | 727 | 18 |
| DC52892 | SAMN32531330 | 2015 | China | Shandong | Meat | MSSA | 7 | CC7 | 2807515 | 767690 | 170929 | 155973 | 815392 | 589 | 18 |
| DC52905 | SAMN32531331 | 2012 | China | Shandong | Rice flour product | MSSA | 7 | CC7 | 2789769 | 706782 | 109820 | 174360 | 1307797 | 590 | 16 |
| DC52912 | SAMN32531332 | 2018/6/15 | China | Heilongjiang | Soy product | MSSA | 7 | CC7 | 2703421 | 1361750 | 127880 | 300380 | 1361750 | 575 | 9 |
| DC52922 | SAMN32531333 | 2018/9/3 | China | Heilongjiang | Frozen drink | MSSA | 7 | CC7 | 2785827 | 846482 | 110994 | 185721 | 1027277 | 819 | 15 |
| DC52928 | SAMN32531334 | 2016/3/22 | China | Yunnan | Rice flour product | MSSA | 7 | CC7 | 2739094 | 602970 | 127829 | 228257 | 874318 | 567 | 12 |
| DC52929 | SAMN32531335 | 2016/6/13 | China | Yunnan | Meat product | MSSA | 7 | CC7 | 2792604 | 319949 | 153486 | 164270 | 637106 | 567 | 17 |
| DC52930 | SAMN32531336 | 2016/7/4 | China | Yunnan | Rice flour product | MSSA | 7 | CC7 | 2787843 | 334303 | 170724 | 185856 | 593086 | 575 | 15 |
| DC52945 | SAMN32531337 | 2017/5/17 | China | Yunnan | Meat product | MSSA | 7 | CC7 | 2785454 | 284577 | 170664 | 163850 | 593519 | 547 | 17 |
| DC52950 | SAMN32531338 | 2017/8/28 | China | Yunnan | Rice flour product | MSSA | 7 | CC7 | 2787335 | 319744 | 109779 | 163960 | 816320 | 505 | 17 |
| DC52953 | SAMN32531339 | 2017/6/26 | China | Yunnan | Meat | MSSA | 7 | CC7 | 2784995 | 553472 | 53332 | 121086 | 603052 | 547 | 23 |
| DC52956 | SAMN32531340 | 2017/8/28 | China | Yunnan | Rice flour product | MSSA | 7 | CC7 | 2740146 | 416410 | 170731 | 228345 | 1162053 | 567 | 12 |
| DC52965 | SAMN32531341 | 2017/6/7 | China | Yunnan | Meat product | MSSA | 7 | CC7 | 2794926 | 292595 | 171887 | 186328 | 637860 | 1516 | 15 |
| DC52966 | SAMN32531342 | 2017/7/5 | China | Yunnan | Meat product | MSSA | 7 | CC7 | 2801097 | 604555 | 186742 | 215469 | 1198993 | 519 | 13 |
| DC52980 | SAMN32531343 | 2017/8/30 | China | Yunnan | Meat | MSSA | 7 | CC7 | 2787622 | 603065 | 109719 | 154867 | 869345 | 507 | 18 |
| DC52998 | SAMN32531544 | 2017/8/25 | China | Yunnan | Rice flour product | MSSA | 7 | CC7 | 2786934 | 603850 | 170911 | 232244 | 913043 | 567 | 12 |
| DC53007 | SAMN32531344 | 2018/6/5 | China | Yunnan | Meat | MSSA | 7 | CC7 | 2789504 | 713913 | 109779 | 185966 | 973547 | 801 | 15 |
| DC53011 | SAMN32531345 | 2018/5/22 | China | Yunnan | Meat | MSSA | 7 | CC7 | 2720422 | 422385 | 170663 | 226701 | 1166198 | 567 | 12 |
| DC53024 | SAMN32531346 | 2018/9/3 | China | Yunnan | Meat product | MSSA | 7 | CC7 | 2791668 | 562451 | 109679 | 139583 | 645487 | 567 | 20 |
| DC53032 | SAMN32531347 | 2018/5/29 | China | Yunnan | Meat | MSSA | 7 | CC7 | 2789902 | 603694 | 186809 | 253627 | 1189984 | 500 | 11 |
| DC53050 | SAMN32531348 | 2018/8/20 | China | Yunnan | Soy product | MSSA | 7 | CC7 | 2720611 | 422385 | 170663 | 226717 | 1166158 | 544 | 12 |
| DC53053 | SAMN32531349 | 2019/6/24 | China | Yunnan | Rice flour product | MSSA | 7 | CC7 | 2750208 | 603487 | 170732 | 229184 | 873875 | 500 | 12 |
| DC53074 | SAMN32531350 | 2018/10/08 | China | Sichuan | Soy product | MSSA | 7 | CC7 | 2762402 | 603291 | 170410 | 197314 | 873519 | 541 | 14 |
| DC53076 | SAMN32531351 | 2016/08/30 | China | Sichuan | Rice flour product | MSSA | 7 | CC7 | 2836503 | 467429 | 95828 | 85954 | 621170 | 529 | 33 |
| DC53082 | SAMN32531352 | 2017/7/17 | China | Sichuan | Meat product | MSSA | 7 | CC7 | 2827321 | 292611 | 94719 | 91203 | 549901 | 561 | 31 |
| DC53124 | SAMN32531353 | 2018/05/18 | China | Sichuan | Fruit product | MSSA | 7 | CC7 | 2794448 | 603789 | 171258 | 214957 | 916885 | 500 | 13 |
| DC53129 | SAMN32531354 | 2016/7/26 | China | Gansu | Meat product | MSSA | 7 | CC7 | 2748166 | 602114 | 170625 | 183211 | 1165762 | 500 | 15 |
| DC53135 | SAMN32531355 | 2016/8/23 | China | Gansu | Rice flour product | MSSA | 7 | CC7 | 2793892 | 294791 | 66406 | 139694 | 571706 | 507 | 20 |
| DC53159 | SAMN32531356 | 2017/5/10 | China | Gansu | Rice flour product | MSSA | 7 | CC7 | 2786747 | 603787 | 109008 | 174171 | 1103997 | 567 | 16 |
| DC53176 | SAMN32531357 | 2018/4/23 | China | Gansu | Rice flour product | MSSA | 7 | CC7 | 2786960 | 604324 | 109007 | 154831 | 810793 | 642 | 18 |
| DC53206 | SAMN32531543 | 2015/11/3 | China | Gansu | Meat | MSSA | 7 | CC7 | 2838828 | 329707 | 109778 | 94627 | 604279 | 509 | 30 |
| DC53212 | SAMN32531358 | 2015/8/10 | China | Gansu | Meat | MSSA | 7 | CC7 | 2792994 | 547007 | 127897 | 79800 | 1035598 | 521 | 35 |
| DC53214 | SAMN32531359 | 2015/8/10 | China | Gansu | Meat | MSSA | 7 | CC7 | 2781050 | 319722 | 127822 | 44855 | 915095 | 503 | 62 |
| DC53243 | SAMN32531360 | 2015/6/1 | China | Gansu | Meat | MSSA | 7 | CC7 | 2750965 | 422482 | 170666 | 250087 | 1166981 | 567 | 11 |
| DC53244 | SAMN32531361 | 2015/6/1 | China | Gansu | Meat | MSSA | 7 | CC7 | 2749227 | 371489 | 170814 | 196373 | 794197 | 522 | 14 |
| DC53284^a^ | SAMN32531362 | 2017/5/3 | China | Hainan | Human | MSSA | 7 | CC7 | 2804801 | 319568 | 54321 | 65227 | 1107514 | 502 | 43 |
| DC53285^a^ | SAMN32531542 | 2017/5/3 | China | Hainan | Human | MSSA | 7 | CC7 | 2789332 | 319495 | 54321 | 103308 | 1107614 | 502 | 27 |
| DC53286^a^ | SAMN32531363 | 2017/5/3 | China | Hainan | Meat | MSSA | 7 | CC7 | 2791872 | 302178 | 54321 | 99709 | 1107677 | 502 | 28 |
| DC53288^a^ | SAMN32531364 | 2017/5/3 | China | Hainan | Baked product | MSSA | 7 | CC7 | 2793759 | 351517 | 54321 | 103472 | 1250858 | 502 | 27 |
| DC53289^a^ | SAMN32531365 | 2017/5/3 | China | Hainan | Baked product | MSSA | 7 | CC7 | 2791112 | 302178 | 54321 | 87222 | 1106977 | 502 | 32 |
| DC53308 | SAMN32531366 | 2015/9/7 | China | Hainan | Meat | MSSA | 7 | CC7 | 2835685 | 652764 | 109665 | 128895 | 769628 | 596 | 22 |
| DC53309 | SAMN32531367 | 2015/6/15 | China | Hainan | Meat | MSSA | 7 | CC7 | 2835805 | 652767 | 78890 | 118159 | 769633 | 592 | 24 |
| DC53311 | SAMN32531368 | 2015/9/7 | China | Hainan | Meat | MSSA | 7 | CC7 | 2833875 | 355677 | 55543 | 104958 | 769626 | 596 | 27 |
| DC53313 | SAMN32531369 | 2015/9/7 | China | Hainan | Meat | MSSA | 7 | CC7 | 2838628 | 397836 | 78962 | 88707 | 1042189 | 610 | 32 |
| DC53314 | SAMN32531370 | 2015/6/15 | China | Hainan | Meat | MSSA | 7 | CC7 | 2833786 | 652769 | 109818 | 123208 | 769635 | 590 | 23 |
| DC53315 | SAMN32531371 | 2015/6/15 | China | Hainan | Meat | MSSA | 7 | CC7 | 2839534 | 292806 | 109779 | 149449 | 599359 | 566 | 19 |
| DC53316 | SAMN32531372 | 2015/6/15 | China | Hainan | Meat | MSSA | 7 | CC7 | 2838866 | 292806 | 109779 | 129039 | 598010 | 511 | 22 |
| DC53318 | SAMN32531373 | 2015/6/15 | China | Hainan | Meat | MSSA | 7 | CC7 | 2839301 | 292806 | 109779 | 149436 | 599413 | 656 | 19 |
| DC53319 | SAMN32531374 | 2015/9/7 | China | Hainan | Meat | MSSA | 7 | CC7 | 2841360 | 292806 | 109779 | 129152 | 599156 | 506 | 22 |
| DC53323 | SAMN32531375 | 2015/8/10 | China | Hainan | Meat | MSSA | 7 | CC7 | 2703970 | 319487 | 54671 | 128760 | 593526 | 507 | 21 |
| DC53328 | SAMN32531376 | 2015/5/25 | China | Hainan | Meat | MSSA | 7 | CC7 | 2909251 | 421609 | 97362 | 24654 | 620873 | 501 | 118 |
| DC53336 | SAMN32531377 | 2015/8/17 | China | Hainan | Aquatic product | MSSA | 7 | CC7 | 2887701 | 324547 | 54320 | 19251 | 1209789 | 501 | 150 |
| DC53349^a^ | SAMN32531378 | 2017/5/3 | China | Hainan | Baked product | MSSA | 7 | CC7 | 2795104 | 604277 | 109779 | 127050 | 814731 | 541 | 22 |
| DC53350 | SAMN32531379 | 2014 | China | Hainan | Soy product | MSSA | 7 | CC7 | 2828275 | 319661 | 101065 | 91234 | 547197 | 621 | 31 |
| DC53362^a^ | SAMN32531380 | 2017/5/3 | China | Hainan | Baked product | MSSA | 7 | CC7 | 2791660 | 604220 | 109779 | 146929 | 814731 | 705 | 19 |
| DC53364 | SAMN32531381 | 2015/9/14 | China | Hainan | Meat | MSSA | 7 | CC7 | 2838138 | 292806 | 109779 | 141906 | 597957 | 1042 | 20 |
| DC53370 | SAMN32531382 | 2015/9/7 | China | Hainan | Meat | MSSA | 7 | CC7 | 2853463 | 292806 | 73408 | 59447 | 598708 | 500 | 48 |
| DC53371 | SAMN32531383 | 2015/8/10 | China | Hainan | Meat | MSSA | 7 | CC7 | 2780697 | 374473 | 128031 | 52465 | 637235 | 505 | 53 |
| DC900018 | SAMN32531384 | 2014/4/9 | China | Jilin | Meat | MSSA | 7 | CC7 | 2736681 | 319523 | 127771 | 228056 | 917606 | 5156 | 12 |
| DC900021 | SAMN32531385 | 2014/4/9 | China | Jilin | Meat | MSSA | 7 | CC7 | 2792657 | 278961 | 70141 | 126938 | 554274 | 1513 | 22 |
| DC900027 | SAMN32531386 | 2014/3/6 | China | Jilin | Rice flour product | MSSA | 7 | CC7 | 2746783 | 440691 | 73390 | 144567 | 569682 | 507 | 19 |
| DC900031 | SAMN32531387 | 2014/4/10 | China | Jilin | Meat | MSSA | 7 | CC7 | 2793264 | 397418 | 109300 | 139663 | 603921 | 706 | 20 |
| DC900039 | SAMN32531388 | 2015/7/6 | China | Jilin | Meat | MSSA | 7 | CC7 | 2793550 | 603338 | 170814 | 214888 | 1209448 | 507 | 13 |
| DC900044 | SAMN32531389 | 2015/7/6 | China | Jilin | Meat | MSSA | 7 | CC7 | 2792905 | 603362 | 170813 | 214838 | 1209511 | 507 | 13 |
| DC900048 | SAMN32531390 | 2016/8/17 | China | Jilin | Rice flour product | MSSA | 7 | CC7 | 2788824 | 284798 | 101058 | 107262 | 397599 | 522 | 26 |
| DC900078 | SAMN32531391 | 2014/5/13 | China | Jilin | Meat | MSSA | 7 | CC7 | 2794145 | 604498 | 170665 | 254013 | 918715 | 5156 | 11 |
| DC900082 | SAMN32531392 | 2014/5/13 | China | Jilin | Meat | MSSA | 7 | CC7 | 2786892 | 397532 | 109779 | 154827 | 604527 | 536 | 18 |
| DC900086 | SAMN32531393 | 2014/7/2 | China | Jilin | Meat | MSSA | 7 | CC7 | 2793657 | 604681 | 170664 | 232804 | 916182 | 593 | 12 |
| DC900087 | SAMN32531394 | 2014/7/2 | China | Jilin | Meat | MSSA | 7 | CC7 | 2783147 | 319438 | 170723 | 154619 | 916346 | 502 | 18 |
| DC900088 | SAMN32531395 | 2014/7/2 | China | Jilin | Meat | MSSA | 7 | CC7 | 2778655 | 397580 | 109781 | 198475 | 603656 | 1011 | 14 |
| DC900107 | SAMN32531396 | 2016/10/8 | China | Jilin | Rice flour product | MSSA | 7 | CC7 | 2817087 | 603999 | 186951 | 90873 | 917319 | 506 | 31 |
| SZ11J01 | SAMN32531397 | 2011/7/20 | China | Guangdong | Rice flour product | MSSA | 7 | CC7 | 2795755 | 707411 | 109835 | 139787 | 850523 | 554 | 20 |
| SZ16J01 | SAMN32531398 | 2016/4/18 | China | Guangdong | Vegetable product | MSSA | 7 | CC7 | 2786256 | 1406565 | 170570 | 174141 | 1406565 | 533 | 16 |
| GCA_001656075.1^b^ | GCA_001656075.1 | 2008 | Germany | NA | Human | MSSA | 7 | CC7 | 2742807 | 2742807 | 2742807 | 2742807 | 2742807 | 2742807 | 1 |
| GCA_003236955.1^b^ | GCA_003236955.1 | 2014 | Italy | Florence | Human | MSSA | 7 | CC7 | 2732783 | 292613 | 54817 | 105107 | 852627 | 1029 | 26 |
| GCA_008934295.1^b^ | GCA_008934295.1 | 2015 | USA | New York City | Human | MRSA | 7 | CC7 | 2889095 | 2844232 | 2844232 | 1444547 | 2844232 | 44863 | 2 |
| GCA_009675705.1^b^ | GCA_009675705.1 | 2013 | Argentina | NA | Human | MSSA | 7 | CC7 | 2785932 | 316457 | 109779 | 60563 | 570654 | 330 | 46 |
| GCA_013307085.1^b^ | GCA_013307085.1 | 2014 | China | Zhejiang | Human | MSSA | 7 | CC7 | 2824297 | 2784836 | 2784836 | 941432 | 2784836 | 4512 | 3 |
| CP051191.1^b^ | CP051191.1 | 2016 | Nigeria | Benin | Human | MRSA | 7 | CC7 | 2880328 | 2880328 | 2880328 | 2880328 | 2880328 | 2880328 | 1 |
| CP051479.1^b^ | CP051479.1 | 2016 | Nigeria | Benin | Human | MSSA | 7 | CC7 | 2793713 | 2793713 | 2793713 | 2793713 | 2793713 | 2793713 | 1 |
| DC51277^c^ | SAMN32531399 | 2006 | China | Guangdong | Meat | MSSA | 943 | CC7 | 2801463 | 703131 | 111595 | 84892 | 848403 | 533 | 33 |
| DC53285^d^ | SAMN32531542 | 2017/5/3 | China | Hainan | Human | MSSA | 7 | CC7 | 2823874 | 2788850 | 2788850 | 1411937 | 2788850 | 35024 | 2 |
| DC53206^d^ | SAMN32531543 | 2015/11/3 | China | Gansu | Meat | MSSA | 7 | CC7 | 2867195 | 2831891 | 2831891 | 1433597 | 2831891 | 35304 | 2 |
| DC52998^d^ | SAMN32531544 | 2017/8/25 | China | Yunnan | Rice flour product | MSSA | 7 | CC7 | 2816898 | 2781852 | 2781852 | 1408449 | 2781852 | 35046 | 2 |
| pDC53285^e^ | SAMN32531542 | 2017/5/3 | China | Hainan | Human | MSSA | 7 | CC7 | 35024 | 35024 | 35024 | 35024 | 35024 | 35024 | 1 |
| ^a^ SFP strains.  ^b^ Strains from NCBI. ^c^ Outgroup.  ^d^ The complete genome of strains.  ^e^ The complete genome of plasmid from SFP DC532285 strain. | | | | | | | | | | | | | | | |
