# Supplementary material for: A food poisoning caused by ST7 Staphylococcal aureus harboring sea gene in Hainan province, China
Source: Front Microbiol. 2023 Mar 16;14:1110720. doi: 10.3389/fmicb.2023.1110720 (PMC10060626; doi:10.3389/fmicb.2023.1110720)
Supplement: Supplementary file 3 [file Table_2.DOCX]

| Differences of resistance genes of ST7 strains from the two clusters in the phylogenetic tree | | | | | |
| --- | --- | --- | --- | --- | --- |
| Gene name | Total* (n=98) | non-SFP (n=91) | No. of strains* | | P^a^ |
|  |  |  | Cluster 1（n=69） | Cluster 2（n=29） |  |
| *APH(3')-IIIa* | 1 (1.0) | 1 (1.1) | 1 (1.4) | 0 (0) | >0.05 |
| *APH(3')-Ia* | 1 (1.0) | 1 (1.1) | 1 (1.4) | 0 (0) | >0.05 |
| *AAC(6')-Ie-APH(2'')-Ia* | 20 (20.4) | 1 (1.1) | 0 (0) | 20 (69.0) | <0.05 |
| *ANT(4')-Ib* | 69 (70.4) | 62 (68,1) | 67 (97.1) | 2 (6.9) | <0.05 |
| *ANT(6)-Ia* | 1 (1.0) | 1 (1.1) | 1 (1.4) | 0 (0) | >0.05 |
| *tetK* | 67 (68.4) | 60 (65.9) | 63 (91.3) | 4 (13.8) | <0.05 |
| *catA* | 1 (1.0) | 1 (1.1) | 1 (1.4) | 0 (0) | >0.05 |
| *ermB* | 20 (20.4) | 20 (22.0) | 0 (0) | 20 (69.0) | <0.05 |
| *ermC* | 25 (25.5) | 25 (27.5) | 25 (36.2) | 0 (0) | <0.05 |
| *lnuA* | 68 (69.4) | 61 (67.0) | 64 (92.8) | 4 (13.8) | <0.05 |
| *msrA* | 1 (1.0) | 1 (1.1) | 1 (1.4) | 0 (0) | >0.05 |
| *mphc* | 1 (1.0) | 1 (1.1) | 1 (1.4) | 0 (0) | >0.05 |
| *blaZ* | 94 (95.9) | 87 (95.6) | 69 (100.0) | 25 (89.7) | >0.05 |
| *lmrS* | 98 (100.0) | 91 (100.0) | 69 (100.0) | 29 (100.0) | \ |
| *norA* | 98 (100.0) | 91 (100.0) | 69 (100.0) | 29 (100.0) | \ |
| ^a^ A P-value<0.05 was considered as statistically significant.  * Values in parentheses are percentages. | | | | | |
