# Supplementary material for: A food poisoning caused by ST7 Staphylococcal aureus harboring sea gene in Hainan province, China
Source: Front Microbiol. 2023 Mar 16;14:1110720. doi: 10.3389/fmicb.2023.1110720 (PMC10060626; doi:10.3389/fmicb.2023.1110720)
Supplement: Supplementary file 4 [file Table_3.DOCX]

| Virulence genes distribution in SFP strains and non-SFP strains | | | | | |
| --- | --- | --- | --- | --- | --- |
| Virulence factor class | Gene^a^ | No. of strains | | χ^2^ | P^b^ |
|  |  | SFP（n=7） | non-SFP (n=91) |  |  |
| Secretion system | *clpP* | 0 | 1 | NA | NA |
|  | *esaG1* | 7 | 91 | NA | NA |
|  | *esaG3* | 7 | 85 | NA | NA |
|  | *esaG4* | 0 | 1 | NA | NA |
|  | *esaG7* | 7 | 89 | NA | NA |
|  | *esaA* | 7 | 91 | NA | NA |
|  | *esaB* | 7 | 91 | NA | NA |
|  | *esaC* | 7 | 91 | NA | NA |
|  | *esaD* | 7 | 91 | NA | NA |
|  | *essA* | 7 | 91 | NA | NA |
|  | *essB* | 7 | 91 | NA | NA |
|  | *essC* | 7 | 91 | NA | NA |
|  | *esxA* | 7 | 91 | NA | NA |
|  | *esxB* | 7 | 91 | NA | NA |
| Iron absorption regulation | *sirA-C* | 7 | 91 | NA | NA |
|  | *sfaA-D* | 7 | 91 | NA | NA |
|  | *sbnA-I* | 7 | 91 | NA | NA |
|  | *htsA-C* | 7 | 91 | NA | NA |
|  | *isdA* | 7 | 89 | 0 | >0.05 |
|  | *isdB* | 7 | 91 | NA | NA |
|  | *isdC* | 7 | 91 | NA | NA |
|  | *isdD* | 7 | 90 | NA | NA |
|  | *isdE* | 7 | 91 | NA | NA |
|  | *isdF* | 7 | 91 | NA | NA |
|  | *isdG* | 7 | 91 | NA | NA |
|  | *isdH* | 7 | 91 | 1.41126 | >0.05 |
|  | *isdI* | 7 | 91 | NA | NA |
| Adherence | *eno* | 7 | 91 | NA | NA |
|  | *srtB* | 7 | 91 | NA | NA |
|  | *sasC* | 7 | 90 | NA | NA |
|  | *atl* | 7 | 90 | NA | NA |
|  | *clfA* | 7 | 90 | 0.323656 | >0.05 |
|  | *clfB* | 7 | 91 | 1.14234 | >0.05 |
|  | *eap/map* | 7 | 91 | 5.084732 | >0.05 |
|  | *ebh* | 7 | 90 | NA | NA |
|  | *ebp* | 7 | 91 | 3.589803 | >0.05 |
|  | *efb* | 7 | 91 | 4.078777 | >0.05 |
|  | *emp* | 7 | 91 | NA | NA |
|  | *fnbA** | 7 | 87 | 20.229266 | <0.001 |
|  | *fnbB** | 7 | 87 | 20.522967 | <0.001 |
|  | *icaA* | 7 | 90 | 0.124562 | >0.05 |
|  | *icaB* | 7 | 90 | 0 | >0.05 |
|  | *icaC* | 7 | 90 | 1.14234 | >0.05 |
|  | *icaD* | 7 | 91 | 0.972214 | >0.05 |
|  | *icaR* | 7 | 87 | 0.940302 | >0.05 |
|  | *sdrC* | 7 | 88 | 6.074263 | >0.05 |
|  | *sdrD** | 7 | 88 | 22.93584 | <0.001 |
|  | *sdrE** | 7 | 87 | 7.994496 | <0.05 |
| Toxin | *lukS_PV** | 7 | 91 | 10264.25512 | <0.001 |
|  | *lukF_PV** | 7 | 91 | 10264.25512 | <0.001 |
|  | *hlb* | 7 | 91 | NA | NA |
|  | *hld* | 7 | 91 | 0.972214 | >0.05 |
|  | *hlgA* | 7 | 91 | 0 | >0.05 |
|  | *hlgB* | 7 | 91 | 0.124562 | >0.05 |
|  | *hlgC* | 7 | 90 | 1.41126 | >0.05 |
|  | *hly/hla* | 7 | 90 | 0.498419 | >0.05 |
|  | *lukD** | 7 | 91 | 45.65083 | <0.001 |
|  | *lukE** | 7 | 90 | 43.892604 | <0.001 |
|  | *sea** | 7 | 3 | 4.055734 | <0.05 |
|  | *selx* | 7 | 91 | NA | NA |
|  | *sel** | 0 | 3 | 13.66045 | <0.001 |
|  | *sec** | 0 | 3 | 5.408349 | <0.05 |
|  | *sep** | 0 | 68 | 177.197526 | <0.001 |
|  | *set12* | 7 | 91 | NA | NA |
|  | *set19* | 7 | 91 | NA | NA |
|  | *set21* | 7 | 91 | NA | NA |
|  | *set22* | 7 | 91 | NA | NA |
|  | *set24* | 7 | 90 | NA | NA |
|  | *set34* | 7 | 91 | NA | NA |
|  | *set39* | 7 | 91 | NA | NA |
|  | *set6* | 7 | 91 | NA | NA |
|  | *set7* | 7 | 91 | NA | NA |
|  | *set8* | 7 | 91 | NA | NA |
|  | *tsst-1** | 0 | 3 | 1.081882 | <0.05 |
| Immune evasion | *adsA* | 7 | 91 | 0.880031 | >0.05 |
|  | *cap5A* | 7 | 91 | NA | NA |
|  | *cap5G* | 7 | 91 | NA | NA |
|  | *cap5M* | 7 | 91 | NA | NA |
|  | *cap8B* | 7 | 91 | NA | NA |
|  | *cap8E* | 7 | 91 | NA | NA |
|  | *cap8F* | 7 | 91 | NA | NA |
|  | *cap8H* | 7 | 91 | NA | NA |
|  | *cap8I* | 7 | 90 | NA | NA |
|  | *cap8J* | 7 | 91 | NA | NA |
|  | *cap8K* | 7 | 91 | NA | NA |
|  | *cap8O* | 7 | 91 | NA | NA |
|  | *cap8P* | 7 | 91 | NA | NA |
|  | *capC* | 7 | 90 | NA | NA |
|  | *capE* | 7 | 91 | NA | NA |
|  | *capG* | 0 | 1 | NA | NA |
|  | *capL* | 7 | 91 | NA | NA |
|  | *chp** | 7 | 15 | 13.573 | <0.05 |
|  | *sbi* | 7 | 91 | 1.237105 | >0.05 |
|  | *scn* | 7 | 91 | 0.805463 | >0.05 |
|  | *spa* | 7 | 90 | NA | NA |
| Enzyme | *aur* | 7 | 91 | 0.805463 | >0.05 |
|  | *geh* | 7 | 91 | 1.41126 | >0.05 |
|  | *hysA* | 7 | 91 | 2.336391 | >0.05 |
|  | *lip* | 7 | 69 | NA | NA |
|  | *nuc* | 7 | 91 | NA | NA |
|  | *sak** | 7 | 85 | 11.929577 | <0.05 |
|  | *splA* | 7 | 91 | NA | NA |
|  | *splB* | 7 | 91 | NA | NA |
|  | *splC* | 7 | 91 | NA | NA |
|  | *splD* | 7 | 91 | NA | NA |
|  | *splE* | 7 | 91 | NA | NA |
|  | *splF* | 7 | 91 | NA | NA |
|  | *sspA* | 7 | 91 | 0.888366 | >0.05 |
|  | *sspB** | 7 | 67 | 248.63676 | <0.001 |
|  | *sspC* | 7 | 91 | 0.55841 | >0.05 |
| ^a^ The differences between the non-food poisoning isolates the data of a published manuscript (43), in which genome of 10,288 S. aureus strains were included.  ^b^ A P-value<0.05 was considered as statistically significant. * There were statistically significant differences in gene carriage between the non-food poisoning isolates in this study and the published manuscript data. | | | | | |
