# Supplementary material for: A food poisoning caused by ST7 Staphylococcal aureus harboring sea gene in Hainan province, China
Source: Front Microbiol. 2023 Mar 16;14:1110720. doi: 10.3389/fmicb.2023.1110720 (PMC10060626; doi:10.3389/fmicb.2023.1110720)
Supplement: Supplementary file 5 [file Table_4.DOCX]

| PCR primers in this study | | | |
| --- | --- | --- | --- |
| **Primer name** | **Gene name** | **Primer sequences (5'–3’)^a^** | **Amplicon size (bp)** |
| Restriction Modification (R-M) genes | *hsdM1* | F:ACCCTGGGAATCTCAACTCT | 1678 |
|  |  | R:AACATCGCAACATAACCAAA |  |
|  | *hsdM2* | F:TCTAAGATACTGGCGAAGATTGT | 933 |
|  |  | R:GTATTACTCATCTTTCAACACCC |  |
|  | *hsdS1* | F:AGAAATCGCAGAAGTTGAACAAG | 1591 |
|  |  | R:TACGCAATACGAACAATCCTCAT |  |
|  | *hsdS2* | F:TCCTAAACTGCCAATAACTAAACTC | 1750 |
|  |  | R:ACGATTACAACCTAAACATACCG |  |
| Primers for confirming closed genome | DC53285 | F:TGTGGGAAGACATAAAAGAAAAAG | 332 |
|  |  | R:CATCAAAATATACATCATCTCGTG |  |
|  | DC53206 | F:TTTTGTTCTCTTCAGTTGGTTTAT | 386 |
|  |  | R:AGTGAGTCTTAGTGCTCATTTGTG |  |
|  | DC52998 | F:TGTGGGAAGACATAAAAGAAAAAG | 332 |
|  |  | R:CATCAAAATATACATCATCTCGTG |  |
| Primers for confirming closed plasmid | pDC53285 | F:CGCACTCGTTTGTTTATATATTTTTTG | 319 |
|  |  | R:ATTATTTTTATCTACCCATTCATTTTG |  |
| ^a^ The primers were designed by this study. | | | |
